# Low resting metabolic rate is associated with greater lifespan because of a confounding effect of body fatness

**DOI:** 10.1007/s11357-014-9731-3

**Published:** 2014-12-11

**Authors:** Luiza C. Duarte, John R. Speakman

**Affiliations:** 1 Institute of Biological and Environmental Sciences, University of Aberdeen, Aberdeen, Scotland AB24 2TZ UK; 2 Institute of Genetics and Developmental Biology, Chinese Academy of Sciences, 1 West Beichen Road, Chaoyang, Beijing, People’s Republic of China

**Keywords:** Metabolic rate, Lifespan, Body composition, Fat mass, Oxidative damage, Mice

## Abstract

A negative association between resting metabolic rate (RMR) and lifespan is the cornerstone of the rate of living and free-radical damage theories of aging. Empirical studies supporting a negative association of RMR to lifespan may arise from the correlation between RMR and both daily energy expenditure (DEE) and thermoregulatory activity energy expenditure (TAEE). We screened 540 female mice for higher and lower DEE and measured RMR in the resulting 324 (60 %). We then selected 92 mice in which there was no link between residual from the regression of RMR against body mass (BM) and residual of DEE against BM to separate the effects of these traits. Lifespan was not significantly related to body mass, DEE and TAEE, but significantly negatively related to RMR. Fat-free mass (FFM) and fat mass (FM) were both significantly positively related to RMR. After removing the effect of FFM on RMR, the association between RMR and lifespan remained significantly negative; however, after statistically removing the effect of FM on RMR, the significant association between RMR and lifespan disappeared. We conclude that the negative association between RMR and lifespan is primarily due to the effect of FM, with FM positively related to both RMR and mortality and hence RMR negatively to lifespan. In 40 additional screened mice, greater FM was also associated with greater oxidative damage to DNA.

**Electronic supplementary material:**

The online version of this article (doi:10.1007/s11357-014-9731-3) contains supplementary material, which is available to authorized users.

## Introduction

The idea that there is a negative relationship between the resting metabolic rate (RMR) and lifespan is at least 100 years old and probably originated with Rubner who observed in 1908 that larger, longer lived animals had lower metabolic rates—and in particular that the product of their metabolism (per gram) and lifespan was essentially constant (Rubner 1908). These data formed a cornerstone of the rate of living theory (Pearl 1928) and later the free-radical damage theory (Beckman and Ames 1998) of aging, both of which proposed that high rates of metabolism are detrimental to longevity. The free-radical damage theory suggests that this is because high metabolic rates lead to greater oxidative stress. Among ectotherms, the association receives strong support. Reducing body temperature, which lowers metabolism, increases lifespan (Van Voorhies and Ward 1999). Among endotherms, however, the data are more confusing. For example, it was widely believed that the effect of caloric restriction (CR) on lifespan might be traced to lowering of mass-adjusted metabolic rate. However, after a transient initial response, CR does not necessarily lead to reduced metabolic rate (Mccarter et al. 1985; Ramsey and Hagopian 2006; Raman et al. 2007; Selman et al. 2005; Speakman and Mitchell 2011). More recent analyses of the inter-specific relationship of mass-adjusted RMR to lifespan, based on substantially more data than were available to Rubner, suggest that there is no association of lifespan to resting metabolism once the confounding effects of body size are taken into account (Speakman 2005). Moreover, comparisons across major classes reveal a positive association. Birds, for example, combine exceptional longevity relative to mammals with elevated resting metabolic rates (Holmes and Austad 1994; Holmes et al. 2001; Furness and Speakman 2008) and exceptional resistance to oxidative stress (Barja et al. 1994; Ogburn et al. 2001); bats also combine high metabolic rates with long lifespans, while marsupials which have generally lower resting metabolic rates than the eutherian mammals also generally live shorter lives (Austad and Fischer 1991). Within species, it has been shown in mice (strain MF1) that those with higher metabolic rates live longer (Speakman et al. 2004). In addition, experimental elevations of metabolism in rodents by forcing them to exercise, or exposing them to cold, do not produce the anticipated reductions in lifespan (Selman et al. 2008; Holloszy 1993; Vaanholt et al. 2010). Despite this weight of evidence against the link between resting metabolic rate and lifespan, there are observations that support such an association. In particular, it was recently observed that high basal metabolic rate in the Baltimore longitudinal study of aging based on over 3000 individuals was a risk factor for increased mortality (Ruggiero et al. 2008; Schrack et al. 2014). Similar results were observed in Pima Indians (Jumpertz et al. 2011), and responses to CR in humans appear to include sustained reductions in RMR (Redman et al. 2009; Weyer et al. 2000; Martin et al. 2007). Finally, elevated RMR has been linked to greater oxidative DNA damage across rat strains that differ in longevity (Greenberg et al. 2000).

One problem with diagnosing the effects of RMR on lifespan is that RMR is typically associated with other features of energy balance. For example, in our previous study of the effects of metabolism on lifespan in mice (Speakman et al. 2004), the mice that lived longest not only had elevated resting metabolism but also increased total metabolism and increased expenditure on physical activity. While we also observed increased levels of mitochondrial proton leak linked to activation of UCP3 and ANT consistent with the “uncoupling to survive” hypothesis (Brand 2000), it remains possible that the longer lifespan of these mice was a consequence of their elevated expenditure on physical activity. Moreover, in free-living humans, the potential links between metabolism and personality (Careau et al. 2008) may mean individuals with greater RMR engage in more risky health-related behaviours (for example, most individuals in the study of Pima Indians died from alcohol-related incidents; Jumpertz et al. 2011).

Evaluating the independent effects of RMR on lifespan is difficult because RMR cannot be experimentally manipulated independent of the other components of energy expenditure. For example, giving animals thyroxine experimentally elevates RMR, but it also leads to greater food intake and physical activity. To evaluate the effects of RMR on lifespan independent of these other factors, we used a different approach. This approach involved measuring a large number of animals for the relevant parameters and then choosing a subset of these animals where the correlations between the traits in question are not significant. We chose the outbred MF1 strain because of the known prior association between metabolism and lifespan in this strain (Speakman et al. 2004) and our extensive prior knowledge of the features of its energy balance. By examining the relationship between RMR and longevity in this special subset of animals, we could be certain that any effect of RMR had not come about because of correlated effects of the other metabolic components.

The selection procedure was as follows (more details are available on the electronic supplementary materials). We screened an initial population of 540 individually housed MF1 mice aged 10 weeks for daily food intake as a proxy for daily energy expenditure (DEE) (see “Materials and methods”) and retained 60 % (*n* = 324) of these for further screening of RMR. This initial screening involved plotting DEE against body mass and fitting a linear regression to the data using the least squares fit procedure. We then calculated the residuals to the fitted regression line and selected for inclusion those individuals with the highest and lowest residuals (top and bottom 30 %). Both variables were tested for normality using the Kolmogorov–Smirnov test prior to fitting the regression model, and the generated residuals and the plotted data were examined for any indication of non-linearity that might invalidate making a linear fit. Because a fitted regression is most strongly influenced by the data that are in close proximity to the regression line, eliminating 40 % of data that sat closest to the fitted regression line reduced the significance of the relationship between DEE and body mass. This procedure therefore reduced the covariance due to body mass differences between the individuals, which is a major factor influencing metabolic rates and food intake. In the selected 324 individuals, we measured the RMR based on measurements of oxygen consumption in the thermoneutral zone using indirect calorimetry (for details, see Supplementary material and methods). For these individuals, we used the same procedure described above for food intake to remove the effect of body mass on RMR by plotting RMR against body mass (BM). We fitted a least squares regression to these data and then calculated the residual RMRs, retaining the 25 % highest and 25 % lowest residual RMRs. For this sample (*n* = 162), we plotted the residual RMR (to body mass) against the residual DEE (to body mass) and fitted a regression to the data. We sequentially eliminated 30 more individuals from this regression until it was no longer anywhere near to significance (*P* > 0.1). In total, we eliminated 192 individuals in this process. We retained a total of 132 individuals in which there was no association between residual RMR and the residual DEE estimated from the food intake. In this manner, we could then explore the association between RMR and lifespan uncontaminated by the covariance effects of RMR with both DEE and BM. We calculated the difference between RMR and DEE and called this the thermoregulatory and activity energy expenditure (TAEE). In 40 of these animals, we sought associations between energetic measures and measures of oxidative stress, while the remaining 92 were used in lifespan studies. There was a significant repeatability of body mass, RMR and residual RMR when comparisons were made between measurements at the screening age of 10 weeks and when they were 9–11 months old (Duarte et al. 2010). Association analyses were performed using data for animals at the older age for both longevity and assays of damage/protection experiments.

## Materials and methods

All experiments were authorised by a local ethical review committee and carried out under UK Home Office regulations. All animals were maintained in specific pathogen-free facilities monitored using sentinel animals analysed at regular intervals. During the study, we had sporadic records of mouse parvovirus and *Klebsiella* sp. in the sentinels, but not from the rooms where these animals were kept.

**Fig. 1:**
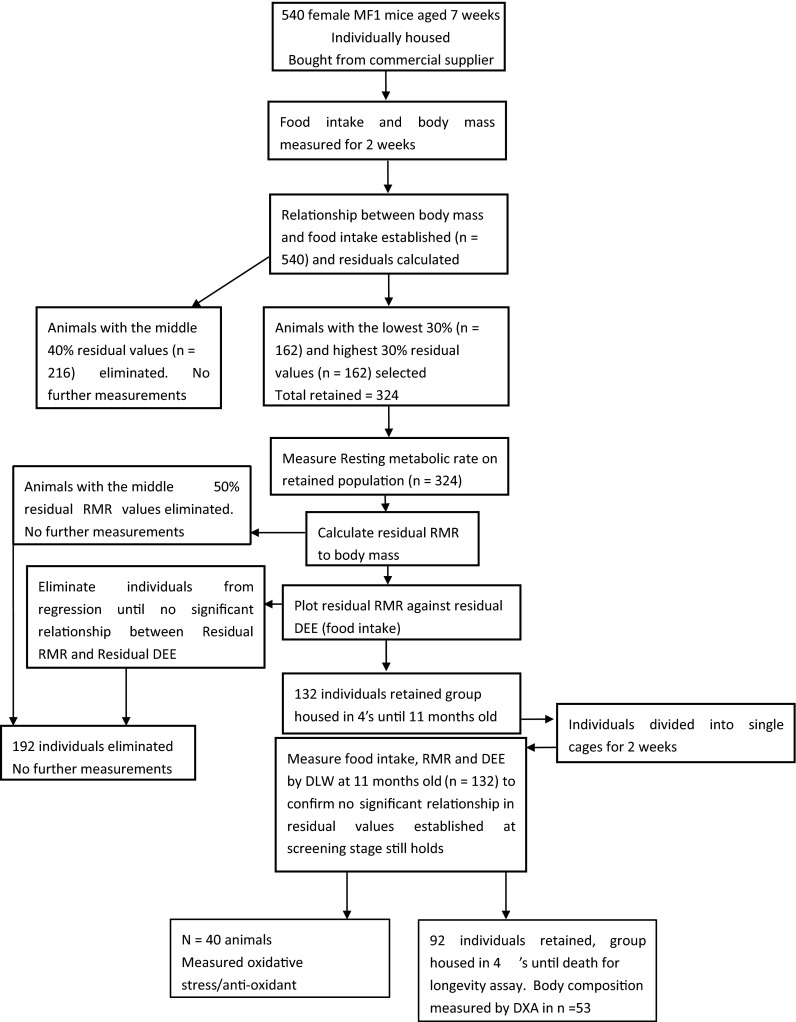
Steps followed for mice screening and the respective sample size in each step

Our aim in this work was to screen a large population of mice for aspects of their energy balance and then select from this population a subset where the covariances between components of balance were not significant. The overall selection process is summarised by the flow diagram in Fig. 1 (detailed material and methods available as electronic supplementary material—ESM).

A total of 540 virgin female mice (*Mus musculus* L.: outbred strain MF1) were purchased from Harlan UK Ltd., Oxon, UK, at 7 weeks of age, in six separate batches. After 1 week of acclimation, they were housed individually in shoebox cages (440 × 120 × 130 mm) with sawdust and paper bedding. The lights were maintained on a 12:12-L/D photoperiod (lights on at 0700 hours), and the ambient temperature was regulated at 21 ± 1 °C. Animals received pellet rodent food [CRM (P), Special Diet Services, BP Nutrition, UK] and water ad libitum. After the acclimation period, we estimated the total DEE by measuring food intake over a 2-week period at 8–10 weeks of age. DEE and BM (per batch) were tested for normality using Kolmogorov–Smirnov tests and were both found to be normally distributed. Estimated DEE from food intake was positively related to body mass for each batch. Analysis of the distribution of residuals in relation to BM and visual inspection of the plot did not indicate the relationship to be non-linear. To minimise the influence of body mass effects, we selected 60 % of the individuals (*n* = 324) that had the highest 30 % and lowest 30 % residual intakes relative to the regression of intake on body mass. We eliminated the animals with the central 40 % residual values (*n* = 216). In the selected 324 individuals, we measured the RMR based on measurements of oxygen consumption in the thermoneutral zone using indirect calorimetry (for details, see Supplementary material and methods). For these individuals, we used the same procedure described above for food intake to remove the effect of body mass on RMR. That is, we tested both variables for normality using Kolmogorov–Smirnov tests, plotted the variables against each other and fitted a least squares linear regression. Visual inspection of the plot and analysis of the residuals in relation to BM revealed no indication of non-linearity in the data. We then eliminated the middle 50 % of the data with the smallest residual values, i.e. retaining the 25 % highest and 25 % lowest residual RMRs from the relationship between RMR and body mass. For this sample (*n* = 162), we plotted the residual RMR (to body mass) against the residual DEE (to body mass) and fitted a least squares regression to the data. We sequentially eliminated 30 more individuals from this regression until it was no longer near to significance (*P* > 0.1). In total, we eliminated 192 individuals in this process. We retained a total of 132 individuals in which there was no association between residual RMR and the residual DEE estimated from the food intake.

After screening, mice were housed in large cages (370 × 255 × 190 mm) in groups of four animals per cage. When the animals were aged 11 months, they were transferred to individual cages (440 × 120 × 130 mm) and kept for 2 weeks for acclimation. They then had their individual food intake, RMR and DEE measured using the doubly labelled water technique. We performed these measurements to confirm that there was still no relationship between the traits that had been used to select them when they had been aged around 4 months. In addition, we used an independent measure of daily energy expenditure (doubly labelled water) because of the known potential problems with equating food intake and total daily energy expenditure. Food intake and respirometry measurements were performed in the same way as described for the screening stage. Ninety two of these individuals were subsequently involved in a longevity screen and were kept in groups of four animals per large cage. When animals died, the groups were joined to form new groups of four to ensure that lifespan was not correlated with group size. The remaining 40 animals were used in laboratory assays for measurements of oxidative stress and antioxidant protection.

This process of selection generates a subset of animals where there was no relationship between the residual RMR and the residual DEE at the age of about 4 months, which was confirmed when the animals were 11 months old using an independent method to measure the DEE. There was similarly no relationship at this age between RMR and TAEE. This selection process was the major strength of the current study because it uniquely allowed us to diagnose the effect of RMR on longevity uncontaminated by the covariance of RMR with either total DEE or TAEE.

### Body composition measurements

All animals used for laboratory assays on aging markers (*n* = 40) and a subsample of 53 animals kept for longevity had their body composition measured using dual-energy X-ray absorptiometry DXA instruments GE (previously Lunar) PIXImus2 Series Densitometers installed with software version 1.46.007 (GE Medical Systems Ultrasound and BMD, Bedford, UK). DXA scans and analyses were carried out as instructed by the manufacturer. Mice were weighed and then anaesthetised by 3.5 % isoflurane (NetTech, UK) inhalation for the duration of the X-ray scanning (~3 min). Lunar PIXIMUS 2.10 software was used to calculate total lean mass, total fat mass, total bone mineral density and total bone mineral content in the region of interest (defined as the subcranial body, as recommended by the manufacturer) using a previously described protocol (Johnston et al. 2005). DXA measures the fat mass (FM) component. Fat-free mass (FFM) was obtained by subtracting FM from body mass. We called fatness the residual obtained by the regression of FM against body mass.

### Statistical analysis

For statistical analysis, we used Minitab 15 and SPSS 19 software. A value of *P* < 0.05 was considered statistically significant. We tested all the variables for normality before performing parametric tests using the Kolmogorov–Smirnov test. We detected associations between variables using least squares linear regression analyses. Linearity was confirmed in the relationships by plotting the residuals against the predictor variables and by visual inspection of the plots. We also analysed the data for significant outliers using Cook’s distance but did not detect any data with undue influence that might warrant elimination. We tested differences in lifespan between groups using *t* tests, and Kaplan–Meier mortality curves were constructed to evaluate differences in mortality, determined using the log-rank test. Associations between measures of oxidative stress, body composition and energy expenditure parameters were evaluated using correlation. The significance level for these tests was adjusted to account for multiple testing using the Bonferroni procedure.

## Results

**Fig. 2:**
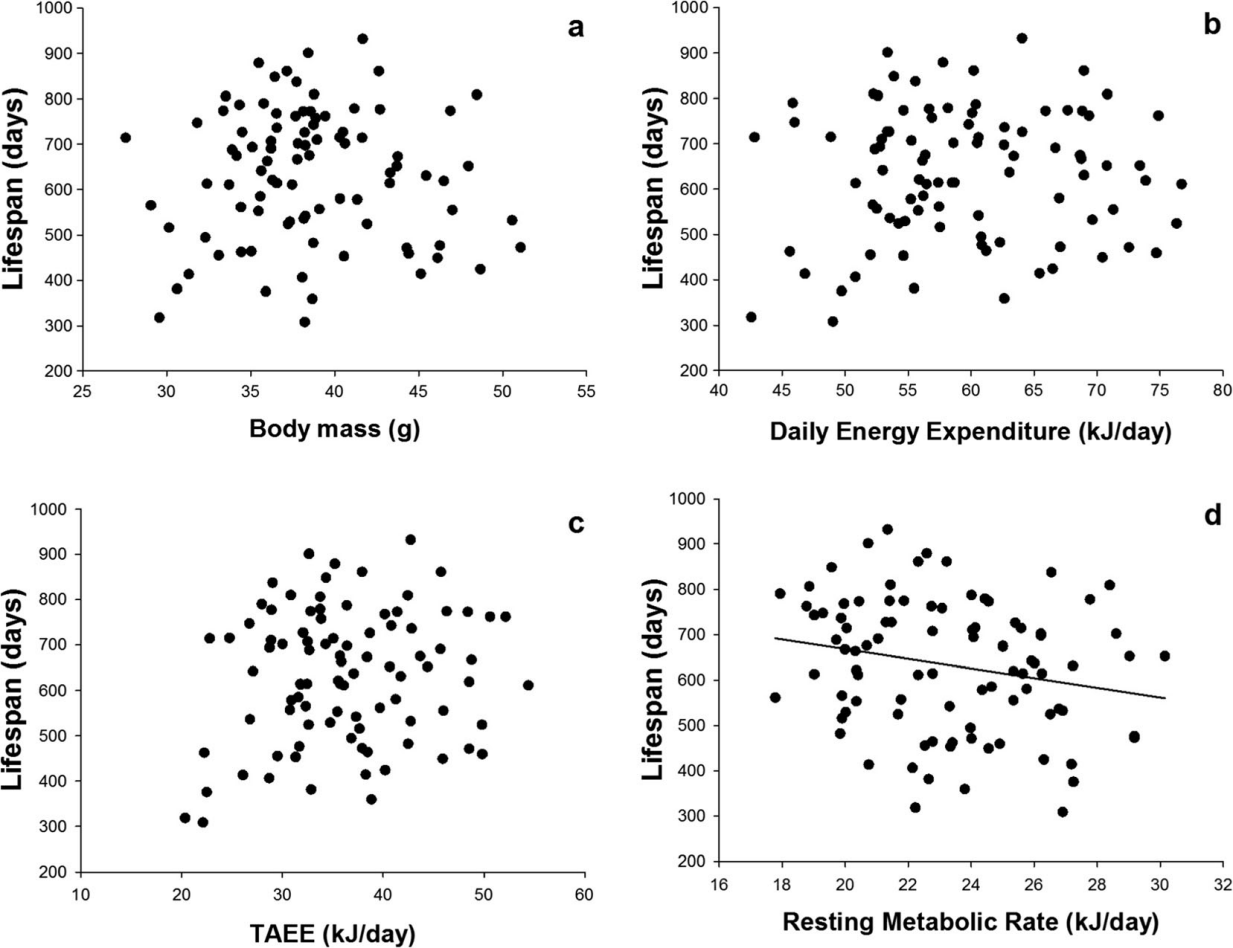
Lifespan correlations. Relationships between individual lifespan of 92 female MF1 mice and body mass (**a**) daily energy expenditure (DEE) (**b**), thermoregulatory activity energy expenditure (TAEE) (**c**) and resting metabolic rate (RMR) (**d**). Only the relationship in **d** was significant (see text for statistics)

**Fig. 3:**
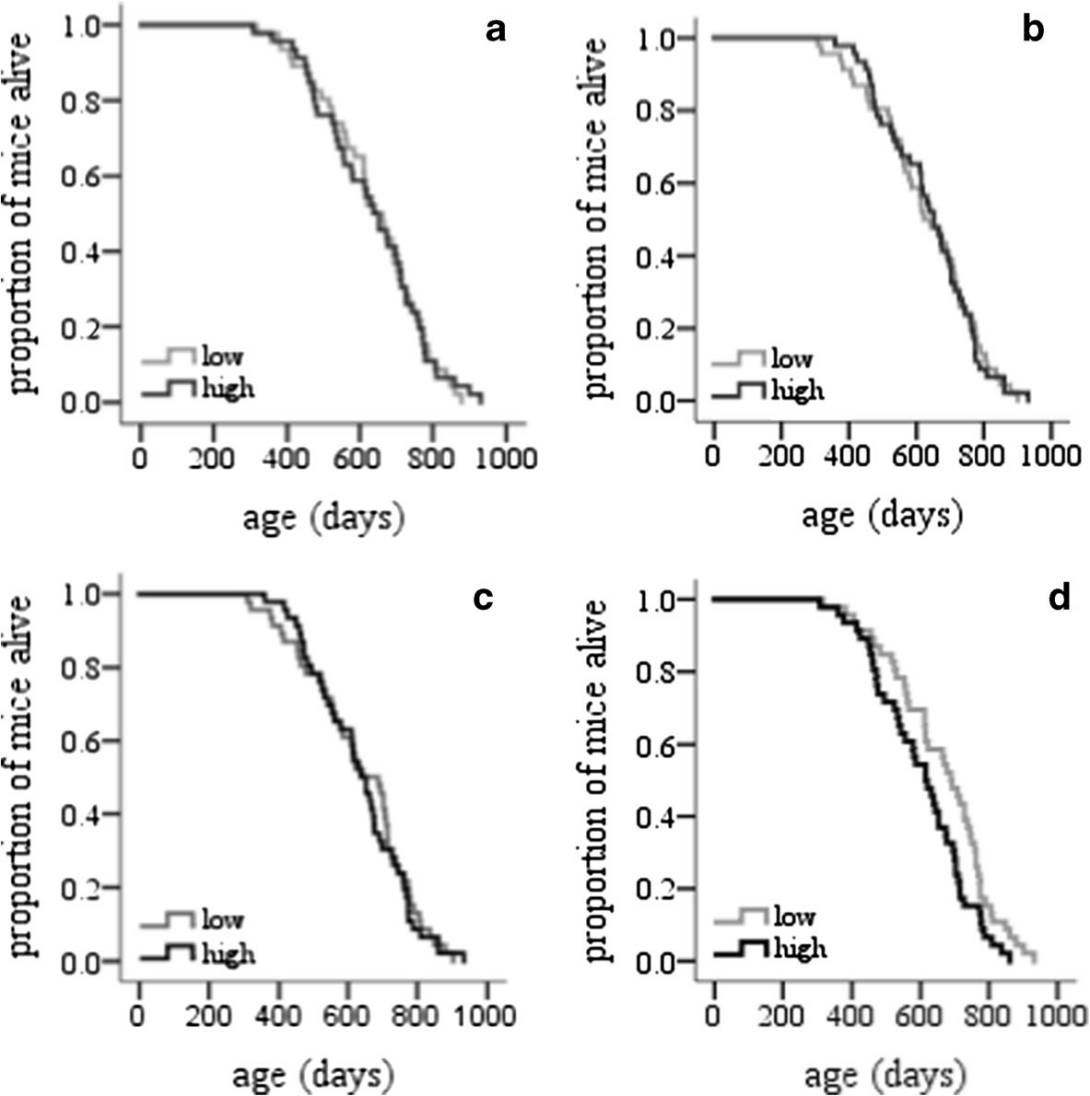
Survival curves. Cumulative survival curve (Kaplan–Meier survival plot) of female MF1 mice. The cumulative survival rate was plotted against age in days. Log-rank test was performed to compare high and low body mass (**a**), daily energy expenditure (**b**), thermoregulatory activity energy expenditure (**c**) and resting metabolic rate (**d**) (see text for statistics)

In the selected sample of 92 individuals that were retained for lifespan analysis, there was no significant relationship between lifespan and body mass (least squares linear regression analysis (LSR): *F*_1,90_ = 0.02, *P* = 0.90; *b* = −0.4; Fig. 2a), lifespan and DEE (LSR: *F*_1,90_ = 0.33, *P* = 0.57; *b* = 1.1; Fig. 2b) or lifespan and TAEE (LSR: *F*_1,90_ = 2.11, *P* = 0.15; *b* = 2.9; Fig. 2c). However, there was a significant negative relationship between RMR and lifespan (LSR: *F*_1,90_ = 4.52, *P* = 0.036; *b* = −10.7; Fig. 2d). We sorted the data set for each variable from the lowest to the highest values of BM, DEE, TAEE and RMR and divided them into two groups for each trait: high and low. We then plotted mortality curves for the divided data and analysed the data in two ways: first comparing the mean lifespan of the two groups using *t* tests and then analysing the mortality rates using the Kaplan–Meier analysis. These results corroborate those obtained by regression analysis on the individual values. There was no significant difference in lifespan between animals with low and high body mass (mean SE lifespan, 635 ± 21 and 630 ± 22 days, respectively; *t* test, *P* = 0.87). Mortality rate comparisons performed using Kaplan–Meier analysis of survival revealed no difference between the two groups (log-rank Mantel–Cox *χ* ^2^, *P* = 0.86; *n* = 92; Fig. 3a). There was no significant difference in lifespan between animals with low and high DEE (mean SE lifespan, 628 ± 22 and 637 ± 20 days, respectively; *t* test, *P* = 0.77) with no difference in mortality rate between the two groups of DEE (log-rank *χ* ^2^, *P* = 0.96; Fig. 3b; *n* = 92). Also, we found no difference between mean lifespan for comparison between low and high TAEE animals (mean ± SE lifespan, 632 ± 23 and 633 ± 20 days, respectively; *t* test *P* = 0.97) and no difference in mortality rate between the two groups (*χ* ^2^, *P* = 0.76; Fig. 3c; *n* = 92). The comparison between RMR longevity curves confirmed that animals with low RMR (mean ± SE lifespan, 662 ± 22 days) lived longer than mice with high RMR (603 ± 20 days; *t* test: *P* = 0.047) by about 10 %. Kaplan–Meier analysis of survival revealed a significant difference in mortality rate between the two groups (log-rank Mantel *χ* ^2^ = 4.9, *P* = 0.027; Fig. 3d; *n* = 92).

**Fig. 4:**
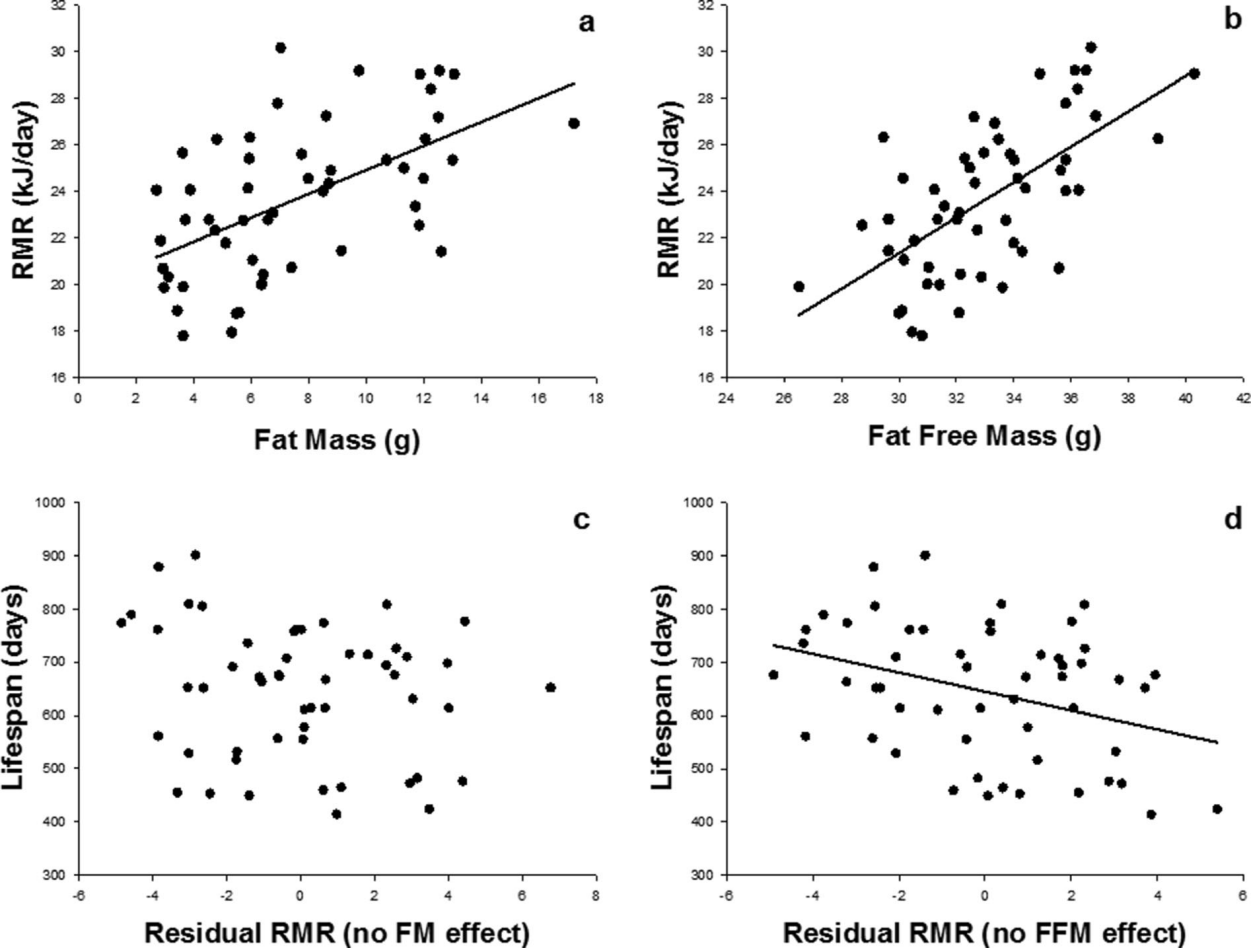
Effect of fat mass and fat-free mass on resting metabolic rate (*RMR*) and lifespan. Relationships between resting metabolic rate and FM (**a**) and FFM (**b**) were both significant. The respective residuals from these regression analyses resulted in the residual resting metabolic rate with no fat mass effect, which was not correlated with lifespan (**c**) and the residual resting metabolic rate with no fat-free mass effect which was significantly correlated with lifespan (**d**) (see text for details (*n* = 53))

**Fig. 5:**
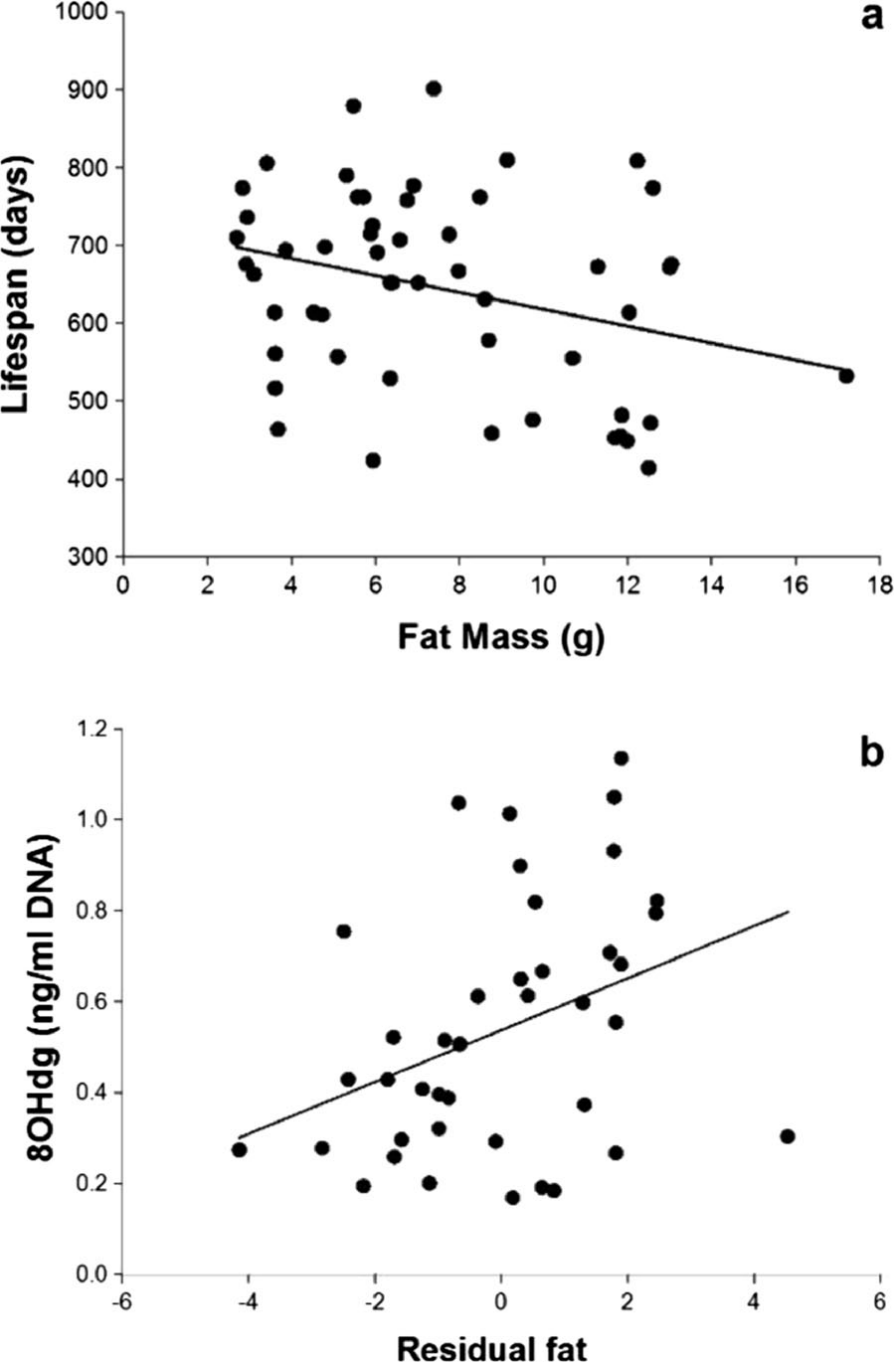
Effect of body fatness on lifespan and DNA damage. Negative and significant relationship between body fatness (g fat) and lifespan (days) (*F* = 5.32, *P* = .025, *R* ^2^ = 0.1, *n* = 53) (**a**). Positive and significant relationship between DNA damage measured by 8-OHdG concentration and body fatness obtained from a regression between fat mass and body mass in grams (*F* = 5.6, *P* = 0.02, *R* ^2^ = 0.13; *n* = 40) (**b**) (see text for details)

RMR is known to be affected not only by variations in body mass, an effect we also confirmed (LSR: *F*_1,90_ = 58.0, *P* < .001, *R* ^2^ = 0.39, *b* = 0.39), but especially by differences in body composition. Fat-free mass (FFM), which accounts for the larger part of body mass, is directly related with RMR, and fat mass (FM) has a smaller but similarly positive contribution to RMR (Johnstone et al. 2005; Kaiyala et al. 2010; Tschöp et al. 2012), possibly mediated in part via an effect of leptin (Kaiyala et al. 2010). We evaluated body composition by measuring FM and FFM in a subsample of mice (*n* = 53). In this sample, the negative relationship between lifespan and RMR was confirmed (LSR: *F*_1,51_ = 6.3, *P* = 0.015; *R* ^2^ = 0.11, *b* = −12.8). To discriminate the effect of FM and FFM on RMR and consequently their putative effects on lifespan, we used the residuals from two separate analyses. In the first, we performed a regression analysis of RMR on FM (LSR: *F*_1,51_ = 23.47, *R* ^2^ = 0.32, *P* < .001; *b* = 0.51, SE_*b*_ = 0.11; Fig. 4a) and calculated the residual, hereafter called “RMR without FM effect”. The second involved a regression of RMR on FFM (LSR: *F*_1,51_ = 35.24, *R* ^2^ = 0.41, *P* < .001; *b* = 0.76, SE_*b*_ = 0.13; Fig. 4b), and the calculated residual was called the “RMR without FFM effect”. The “RMR without FM effect” was not significantly related with lifespan (LSR: *F*_1,51_ = 1.9, *P* = 0.17, *b* = −9.0; Fig. 4c), while “RMR without FFM effect” remained negatively and significantly related with lifespan (LSR: *F*_1,51_ = 7.2, *P* = 0.01, *R* ^2^ = 0.12, *b* = −17.7; Fig. 4d). This analysis indicated that the negative relationship between RMR and lifespan was due to the association between RMR and body fat, as when the effect of FM on RMR was statistically removed, the effect on lifespan disappeared. If the effect of RMR is due to an effect of body fat, then we would anticipate that there would be an effect of fat tissue mass on longevity, but no effect of lean tissue mass. This was indeed the case (Fig. 5a: LSR: *F*_1,51_ = 5.32, *P*=0.02; *R* ^2^ = 0.1, *b* = −10.9), but there was no effect of fat-free mass on longevity (LSR: *F*_1,51_ = 0.46, *P*=0.5, *b* = −4.38: plot not shown). We repeated the analysis using soft lean tissue mass obtained by excluding bone from fat-free mass, and we got the same results (not shown here).

The sample size in this relationship differs from those in Fig. 2d because only a subset of the animals had their body composition measured by DXA (see “Materials and methods”).

We also measured oxidative stress and antioxidant protection in a sample of animals not included in the longevity measurements (*n* = 40). The relationship between body mass and markers of oxidative stress [protein carbonyl; DNA damage—by concentration of 8-hydroxy-2′-deoxyguanosine (8-OHdG); determination of reactive oxygen metabolites (d-ROMs)] was significant only for DNA damage (Table 1; *n* = 40). Of the antioxidant enzyme activities (SOD, catalase and GPx) there was a significant negative relationship with body mass for GPx; no significant correlation was found for the antioxidant adsorbent test (OXY) (Table 1). Due to the significant relationships with body mass, we explored the relationship between oxidative stress and antioxidant protection and RMR, DEE and FM using the residual values for both independent and dependent variables from regression analyses on body mass. The resulting residual RMR, residual DEE and residual FM (fatness) were not significantly correlated with any of the antioxidant enzyme activities. However, residual FM was significantly positively correlated with oxidative damage to DNA (*F*_1,38_ = 5.7, *P* = 0.02, *R* ^2^ = 0.13, *b* = 0.06; Table 1; Fig. 5b).

**Table 1:** Pearson correlation and *P* values for anti-oxidant barriers and oxidative stress. Glutathione peroxidase (GPx); superoxide dismutase (SOD); protein carbonyls (p.carbonyls); determination of Reactive Oxygen Metabolites (d-ROMs); OXY Adsorbent Test (OXY)

	Body mass	*P*	Residual RMR	*P*	Residual DEE	*P*	Residual fat	*P*
GPx^a^	– 0.33	0.039*	0.08	*0.63*	0.03	*0.86*	0.25	*0.11*
SOD^a^	– 0.26	*0.10*	– 0.03	*0.85*	– 0.08	*0.63*	0.10	*0.52*
Catalase^a^	– 0.01	*0.97*	– 0.01	*0.96*	– 0.04	*0.82*	– 0.15	*0.33*
OXY^a^	– 0.09	*0.57*	0.06	*0.72*	– 0.14	*0.40*	0.08	*0.59*
d-ROMS^b^	0.41	0.009**	– 0.06	*0.69*	– 0.02	*0.9*	– 0.04	*0.79*
p. carbonyl^b^	– 0.27	*0.08*	0.04	*0.82*	– 0.02	*0.9*	0.12	*0.45*
DNA damage^b^	– 0.51	0.001**	0.04	*0.80*	0.17	*0.31*	0.37	0.02*

^a^Antioxidant barriers

^b^Oxidative stress

^*^ *P* < 0.05

**P < 0.01

## Discussion

Contrasting some previous studies (Miller et al. 2002), but corroborating our previous work (Speakman et al. 2004), we did not find any association between individual body mass and lifespan in this sample of animals from the MF1 strain (Fig. 2). When we broke the link between daily energy expenditure and resting metabolic rate using the selection procedure described in “Materials and methods”, we also found no effect of DEE on lifespan but that greater RMR was linked to reduced lifespan. Since there was no effect of overall DEE, but a negative effect of a component of DEE (i.e. RMR), then logically one would anticipate that the remaining component of DEE (i.e. TAEE) should be positively associated with lifespan. This trend was indeed observed (Fig. 2c) but failed to reach statistical significance (*P* = 0.15). Prior studies have also shown positive effects of physical activity interventions on average survival time in rodents (Holloszy; Vaanholt et al. 2010). However, this additional effect need not be solely driven by physical activity as TAEE is contributed to by both physical activity and thermoregulatory effects, the latter of which may include elevated metabolism due for example to uncoupled respiration in brown adipose tissue or changes in mitochondrial efficiency due to other leak-mediated processes such as the adenine nucleotide translocase. Indeed, this may also include non-mitochondrial futile cycles, for example, including sarcolipin-mediated activation of the ryanodine receptors and SERCAs in muscle endoplasmic reticulum (Bal et al. 2012). Taken together, the effects of RMR and TAEE observed here suggest that the previous observations of a positive link between DEE and lifespan (Speakman et al. 2004) in this strain were additionally due to a positive effect of physical activity and other metabolic processes as well as an effect mediated via elevated uncoupling. The complete mechanistic basis of the uncoupling to survive effect we previously detected remains to be clarified at the molecular level.

Uncoupling of respiration does seem, however, to be a valid effect mediating lifespan extension. For example, recent studies in which uncoupling in skeletal muscle was increased by transgenic overexpression of UCP-1 also demonstrated increased lifespan (Miller et al. 2002), confirming an influence of uncoupling on survival. The finding that high RMR was associated with shortened lifespan is consistent with the suggestion from both the rate of living and free-radical theories of aging that higher metabolism is detrimental (Pearl 1928; Beckman and Ames 1998) and consistent with recent studies in humans that linked low RMR to greater lifespan (Ruggiero et al. 2008; Schrack et al. 2014; Jumpertz et al. 2011). Measures of body fat, however, were unavailable in one of the studies of humans (Ruggiero et al. 2008); the method for normalising metabolic rate that was employed (using predicted surface areas) may poorly correct for body composition effects for the other study (Jumpertz et al. 2011), and for the third one, body composition was weakly estimated as it was assessed by measurement from one leg (Schrack et al. 2014). When normalising for the effects of FFM and FM removed the significance of the association between mortality risk and 24 h EE (Jumpertz et al. 2011), a marginally significant effect of RMR still remained. That the variation in lifespan explained by “RMR without FFM effect” was only 12 % is not surprising, considering a multi-factorial variable like lifespan, which is dependent on the complex biological phenomenon of aging. Although our data point the finger at fat mass as causing the relationship between RMR and lifespan, it is important to recognise that this inference was based on the correlation analyses illustrated in Fig. 4 and that FM and FFM are correlated. Using correlations in this context can be difficult because the assumption that the predictor variables are independent is violated. Nevertheless, our data clearly implicate FM more than they do FFM. Studies breaking the link between FM and FFM like what we have done to break the link of RMR to DEE would be ideally performed to confirm this inference.

The positive association of RMR to body fat is well established in both humans (Johnstone et al. 2005, Wouters-Adriaens et al. 2007; Wouters-Adriaens and Westerterp 2008) and mice (Kaiyala, et al. 2010). Moreover, the negative effects of fatness on health are similarly well known, particularly in humans (Yusuf et al. 2005), and appear to act via multiple mechanisms including disrupted insulin signalling (Gabriely et al. 2002) and glucose homeostasis and potentially linked to modulations of oxidative stress which were also confirmed in our study, consistent with previous data associating RMR with elevated oxidative damage (Greenberg et al. 2000). The exact molecular mechanisms mediating these effects remain uncertain, but adipose tissue is well established to produce a range of hormones (adipokines including leptin, adiponectin and interleukins) that may mediate negative (or positive) impacts elsewhere in the body. Both enhanced insulin resistance and alterations in oxidative stress would provide tangible mechanistic links to aging and lifespan based on our existing knowledge. Moreover, reduced body fatness has been implicated as a major contributory factor to the benefits of CR (Speakman and Mitchell 2011; Liao et al. 2011). Thus, a negative link between RMR and lifespan mediated via effects of fatness is consistent with a wide range of previous data and supports the suggestions that variations in metabolism per se are not causally linked with the process of aging—contradicting both the rate of living theory and the free-radical theory of aging.

## Electronic supplementary material

Below is the link to the electronic supplementary material.

ESM 1 (DOC 49 kb)
