# Supplementary material for: Low resting metabolic rate is associated with greater lifespan because of a confounding effect of body fatness
Source: Age (Dordr). 2014 Dec 11;36(6):9731. doi: 10.1007/s11357-014-9731-3 (PMC4262579; doi:10.1007/s11357-014-9731-3)
Supplement: Supplementary file 1 — (DOC 49 kb) [file 11357_2014_9731_MOESM1_ESM.doc]

**Low resting metabolic rate is associated with greater lifespan because of a confounding effect of body fatness**

AGE
Luiza C. Duarte*, John R. Speakman

Institute of Biological and Environmental Sciences

University of Aberdeen, Aberdeen, Scotland, UK AB24 2TZ

*Correspondence to: l.duarte@abdn.ac.uk

Tel: 01224 272879

Fax: 01224 272396

**Electronic supplementary material (ESM)**

**Supplementary material and methods**

*Resting metabolic rate*

Resting metabolic rate (RMRt) was measured using four open-flow respiratory systems set at 30°C, within the thermal neutral zone for these mice (Arch et al. 2006; Johnstone et al. 2005, Duarte et al. 2010). Briefly, all measurements were made during the day between 09:00 and 17:00h (2h after normal lights on and before lights off) and started either in the morning around 09:00h or in the afternoon around 13:30h. Oxygen concentrations were measured continuously, and averaged values were stored every 30s for 180min and RMR was quantified as the oxygen consumption over the lowest 10 consecutive values (5min), excluding periods that included transient drops in the measurements (Duarte et al. 2010). The RMR data (ml O_2 ._min^–1^) were converted to energy equivalents using an oxycalorific value of 21.1J. ml^–1^O_2_ derived from the Weir equation (Weir 1949).

*Daily energy expenditure (DEE)*

DEE was measured using the doubly labelled water (DLW) technique (Speakman 1997; Butler et al. 2004). Day-to-day variability in estimated energy metabolism suggests measurements spanning multiple 24·h periods may give a superior representation of energy metabolism (Speakman and Racey 1987; Speakman 1994; Berteaux et al. 1996). Animals were weighed (±0.01·g) and injected intraperitoneally with approximately 0.15·g of water containing enriched *^18^O* (27.8·atom%) and *^2^H* (15.9·atom%). Syringes were weighed before and after administration (±0.0001·g) to calculate the mass of DLW injected. Blood samples were taken from tail after 1·h of isotope equilibration to estimate initial isotope enrichments (Speakman et al. 1990). A final blood sample was taken approximately 48·h later to estimate isotope elimination rates. Blood samples were immediately heat sealed into glass capillaries and stored at room temperature. Drinking water was collected to estimate the background isotope enrichments (method C) (Speakman and Krol 2005). Capillaries that contained the blood samples were vacuum distilled (Visser and Schekkerman 1999) and water from the resulting distillate was used to produce CO_2_ and H_2_ (Lifson et al. 1955; Visser et al. 2000). The isotope ratios *^18^O*:*^16^O* and *^2^H*:*^1^H* were analysed using gas source isotope ratio mass spectrometry (ISOCHROM μGAS system and IsoPrime IRMS, Micromass, Manchester, UK). We ran three high-enrichment standards each day alongside the samples and corrected all the raw data to these standards to minimise the problems of inlet cross contamination. Initial isotope dilution spaces (mol) were calculated by the intercept method, then converted to g assuming a molecular mass of body water of 18.020 and expressed as a percentage of body mass before injection. We used the intercept method because the actual body water pool estimated by desiccation was more accurately predicted by the intercept approach than by the plateau approach. Final dilution spaces were inferred from the final body mass, assuming the same percentage of body water as measured for the initial dilution spaces. The isotope elimination rate (*k*) was calculated following published methods (Lifson et al. 1955). We used the single-pool model equation 7.17 to calculate the rate of CO_2_ production, as recommended for animals weighing under 1·kg (Visser and Schekkerman 1999; Visser et al. 2000; Lifson et al. 1955). Energy equivalents of the rate of CO_2_ production were calculated using a conversion factor of 24.026·J·ml^-1^·CO_2_, derived from the Weir equation for a respiratory quotient of 0.85 (Weir 1949).

*Dissection*

The animals used in the assays experiments were sacrificed by carbon dioxide inhalation at age 11 months. Dissections were performed at approximately the same time in the afternoons to minimize any diurnal variation in the levels of oxidative damage biomarkers. Blood samples were obtained by cardiac puncture. Following cardiac puncture, mice were immediately dissected to collect tissues, which were promptly frozen in liquid nitrogen. Blood samples were clotted on ice and centrifuged. Serum and frozen tissue were stored at −80°C for later analysis.

*Anti-oxidant enzymes on liver tissue*

Prior to enzyme determinations, liver samples were homogenized (5% wt/vol) in cold 50mM phosphate buffer (pH 7.4), centrifuged at 4000 x g for 20 min at 4°C. The supernatant fraction was used for antioxidant enzyme determinations, which were all determined through spectrophotometer. Enzyme activity, were expressed per milligram of protein. Protein concentration of liver extracts was determined using a Bradford assay (Bio-Rad Laboratories Inc., USA) following supplier’s instructions. Catalase activity was determined by an end point assay specific for tissues (Cohen et al. 1970; Aebi 1980; Selman et al. 2000) with modifications. Briefly, catalase activity of 1% Triton X-100 treated supernatant was determined at 25°C by the disappearance of H_2_O_2_ at 480 nm. Glutathione peroxidase (GPx) activity was indirectly measured by a coupled reaction with glutathione reductase (Paglia, and Valentine 1967; Lawrence and Burk, 1976). Superoxide dismutase (SOD) measurements are based on the ability of the enzyme to inhibit the autoxidation of pyrogallol ([Marklund and](http://www.ncbi.nlm.nih.gov/pubmed?term="Marklund S"%5BAuthor%5D) [Marklund](http://www.ncbi.nlm.nih.gov/pubmed?term="Marklund G"%5BAuthor%5D) 1974). One unit of SOD activity is the amount of enzyme that caused 50% inhibition of pyrogallol autoxidation.

*Protein carbonyl*

Protein carbonyls in liver tissue were measured based on 2,4-dinitrophenylhydrazine (DNPH) following published methods (Levine, et al. 1994). We used the “protein carbonyl assay kit” (Cayman, MI, USA).

*Oxidative stress and anti-oxidant barrier in serum*

The serum concentration of ROMs (Reactive Oxygen Metabolites - primarily hydroperoxides ROOH; marker of early oxidative damage) was measured by *d-ROM*s test, following supplier instructions (Diacron, Grosseto, Italy). The test is based on the concept that the amount of organic hydroperoxides present in serum is related to the free radicals from which they are formed, whose concentration can be determined through spectrophotometer. The values are expressed in U.CARR. (Carratelli Units), where 1 U.CARR = 0.8 mg/L H_2_O_2_.

The serum anti-oxidant capacity was measured by the *OXY-Adsorbent* test, following supplier instructions (Diacron, Grosseto, Italy). The test quantifies by colorimetric determination the ability of the anti-oxidant barrier to oppose the massive oxidative action of hypochlorous acid (HOCl), an oxidant of pathologic relevance in biological systems.

*DNA damage*

DNA samples from liver tissue were isolated based on a previously published protocol (Kakimoto et al. 2002) using salt (NaI) instead of phenol and, thereby can avoid any oxidative damage caused by phenol (Helbock et al. 1998). DNA damage was assessed measuring concentration of 8-hydroxy-2’-deoxyguanosine (8-OHdG) by a competitive Elisa plate (50µl/well) according to the protocol supplied by JAICA (Fukuroi, Japan).

**Supplementary references**

Aebi H (1984). Catalase in vitro. *Methods Enzymol.* **105**, 121–126.

Arch, JRS, Hislop, D, Wang, SJY and Speakman, JR (2006) Some mathematical and technical issues in the measurement and interpretation of open-circuit indirect calorimetry in small animals. *Int. J. Obes.* 30, 1322–1331.

Berteaux D, Thomas DW, Bergeron JM, Lapierre H (1996) Repeatability of Daily Field Metabolic Rate in Female Meadow Voles (*Microtus pennsylvanicus*). *Funct. Ecol.* 10, 751–759.

Butler PJ, Green JA, Boyd IL, Speakman JR (2004) Measuring metabolic rate in the field: the pros and cons of the doubly labelled water and heart rate methods. *Funct. Ecol.* 18, 168–183.

Cohen G, Dembiec D, Marcus J (1970) Measurement of catalase activity in tissue extracts. *Anal. Biochem.* 34, 30–38.

Helbock HJ et al. (1998) DNA oxidation matters: The HPLC–electrochemical detection assay of 8-oxo-deoxyguanosine and 8-oxo-guanine. *Proc. Natl. Acad. Sci. USA* 95, 288–293.

# Lawrence, RA and Burk, RF (1976) Glutathione peroxidase activity in selenium-deficient rat liver. *Biochem. Biophys. Res. Comm*. 71, 952–958.

Levine, R., Williams, JA, Stadman, ER, Shacter, E (1994) Carbonyl assays for determination of oxidatively modified proteins. *Methods Enzymol*. 233, 346–357.

Lifson, N, Gordon, GB, McClintock, R (1955) Measurements of total carbon dioxide production by means of D2O18. *J. Appl. Physiol.* 7, 704-710.

Kakimoto M et al. (2002) Accumulation of 8-hydroxy-2'-deoxyguanosine and mitochondrial DNA deletion in kidney of diabetic rats. *Diabetes* 51, 1588–1595.

# [**Marklund** S,](http://www.ncbi.nlm.nih.gov/pubmed?term="Marklund S"%5BAuthor%5D) [**Marklund, G**](http://www.ncbi.nlm.nih.gov/pubmed?term="Marklund G"%5BAuthor%5D) **(**1974) Involvement of the superoxide anion radical in the autoxidation of pyrogallol and a convenient assay for superoxide dismutase. [***Eur. J. Biochem.***](javascript:AL_get(this, 'jour', 'Eur J Biochem.');) 47, 469–474.

Paglia, DE, Valentine, WN (1967) Studies on the quantitative and qualitative characterization of erythrocyte glutathione peroxidase. *J. Lab. Clin. Med.* 70, 158–169.Selman, C, McLaren, JS Himanka, MJ and Speakman, JR (2000) Effect of long term cold exposure on anti-oxidant enzyme activities in a small mammal. *Free Radic. Biol. Med*. 28, 1279–1285.

Speakman JR et al. (1994) Inter- and Intraindividual Variation in Daily Energy Expenditure of the Pouched Mouse (*Saccostomus campestris*). *Funct. Ecol.* 8, 336–342.

Speakman JR (1997) *Doubly-labelled water: theory and practice*. Kluwer Academic publishers, New York.

Speakman JR, Racey PA (1987) The Equilibrium Concentration of Oxygen-18 in Body Water: Implications for the Accuracy of the Doubly-labelled Water Technique and a Potential New Method of Measuring RQ in Free-living Animals. *J. Theor. Biol.* 127, 79–95.

Speakman JR et al. (1990) Interlaboratory comparison of different analytical techniques for the determination of oxygen-18 abundance.  *Anal. Chem.* 62, 703–708.

Speakman JR, Król EB (2005). Comparison of Different Approaches for the Calculation of Energy Expenditure Using Doubly Labelled Water in a Small Mammal. *Physiol. Bioch. Zool.* 78, 650–667.

Visser GH, Schekkerman H (1999) Validation of the Doubly Labeled Water Method in Growing Precocial Birds: The Importance of Assumptions Concerning Evaporative Water Loss. *Physiol. Biochem. Zool.* 72, 740–749.

Visser GH, Boon PE, Meijer HAJ (2000) Validation of the doubly labeled water in Japanese quail *Coturnix c. Japonica* chicks: is there an effect of growth rate? *J. Comp. Physiol. B* 170, 365–372.

Weir JB (1949) New methods for calculating metabolic rate with special reference to protein metabolism. *J. Physiol. Lond.* 109, 1–9.
